# Electrophysiological activity predicts children's reading ability through orthographic awareness: Evidence from a cross-sectional and longitudinal study

**DOI:** 10.1016/j.dcn.2025.101609

**Published:** 2025-08-29

**Authors:** Man Zhang, Zeping Liu, Xuedi Liu, Pengfei Lu, Li Liu, Taomei Guo

**Affiliations:** a State Key Laboratory of Cognitive Neuroscience and Learning & IDG/McGovern Institute for Brain Research, Beijing Normal University, Beijing, China; b Department of Psychology, School of Humanities and Social Sciences, Anhui Agricultural University, Hefei, China; c Department of East Asian Languages and Cultures, Indiana University, Bloomington, IN, USA

**Keywords:** EEG, Resting-state, N170, Orthographic awareness, Reading development

## Abstract

The relationship between brain activity and reading acquisition has been a research focus in recent years. In the current cross-sectional and longitudinal study, we aimed to investigate whether and how resting-state (rs) and task-state brain electrophysiological activity would predict children’s reading ability. Here, we tracked 73 primary school children’ orthographic awareness, reading ability, and EEG signals during both rest and completed a Chinese character recognition task over two consecutive years. Our analyses reveled these neurophysiological measures (rs-EEG power in theta/delta bands and N170 amplitude) significantly predicted orthographic awareness in both cross-sectional and longitudinal analyses. Mediation analysis revealed that these neurophysiological measures influenced reading ability by affecting children's orthographic awareness. Importantly, age moderated these effects: the predictive effect of rs-EEG power was stronger in younger children and decreased with age, whereas the effect of N170 amplitude showed the opposite pattern, becoming more prominent as age increased. Collectively, these findings indicate that children's reading performance is shaped by age-sensitive brain neurophysiological activity, with orthographic processing potentially serving as a key cognitive mechanism.

## Graphical Abstract

**Figure:**
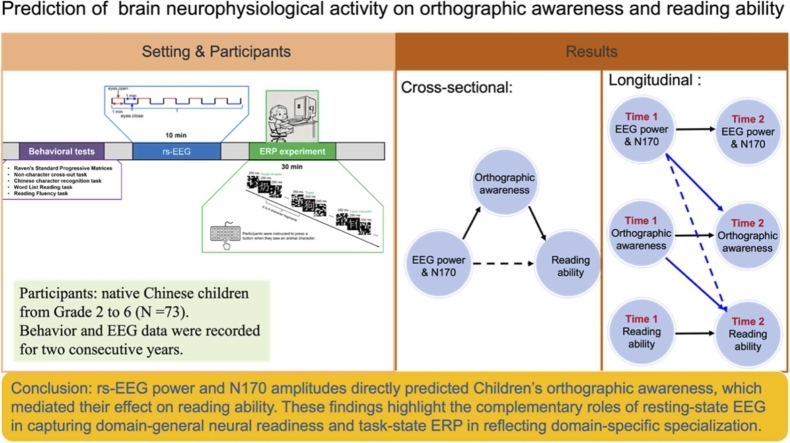

## 1 Introduction

Learning to read is a fundamental skill that supports language development and facilitates knowledge acquisition. Previous research has identified neural markers associated with literacy acquisition (Bach et al., 2013, Brem et al., 2013, Eberhard-Moscicka et al., 2015, Meng et al., 2021), highlighting the brain's capacity to adapt and reorganize in response to learning and reading experiences. While these neural markers provide important information about brain activity associated with reading outcomes, the predictive role of neural indicators in reading performance remains unclear. Additionally, behavioral factors such as orthographic awareness—an aspect of metalinguistic awareness that involves recognizing spelling patterns in written language—are equally critical for predicting Children’s reading development (Conrad et al., 2013, Conrad and Deacon, 2016). Building on this dual perspective, understanding the developmental trajectories of reading-related neural markers and identifying behavioral and neural factors that contribute to individual differences among school children are critical to advance our knowledge of reading development. Thus, the present study drew on a longitudinal design with two measurement points spaced one year apart, focusing on Chinese primary school children aged 7–13—a developmental window characterized by rapid changes in character decoding, reading fluency (Li and Wu, 2015, Liu et al., 2017, Zhang et al., 2024), and neural specialization for print (Tong et al., 2016, Xue et al., 2023, Zhao et al., 2019). We aimed to examine changes in resting-state EEG power and N170 tuning during Chinese character processing, and further investigated whether neural activity could predict orthographic awareness and reading ability. Furthermore, we tested whether orthographic awareness would mediate the relationship between brain activity and reading outcomes, and whether these brain–behavior associations would vary across age to assess the developmental dependency of neural contributions to reading acquisition.

As established in previous ERP studies, the N170 or N1, an early negative component occurring approximately 150–200 ms after stimulus onset, is commonly associated with visual stimulus processing (Bentin et al., 1999, Maurer et al., 2005, Maurer et al., 2005). It is mainly distributed over the occipito-temporal cortex, which is close to the visual word form system (Baker et al., 2007, Brem et al., 2006, Maurer et al., 2005). Most existing studies on the word-related N170 component have demonstrated that stimuli containing orthographic information—such as real words and pseudo-words in alphabetic languages, as well as real characters and pseudo-characters in Chinese—elicit a more negative N170 amplitude compared to visually matched non-orthographic stimuli, such as symbol strings or false characters, a pattern observed in both Chinese and alphabetic languages (Cao et al., 2011, Lin et al., 2011, Maurer et al., 2005, Zhao et al., 2012). Consequently, researchers regard it as the first ERP component indicative of the orthographic processing that occurs during reading (Bentin et al., 1999, Maurer et al., 2005, Maurer et al., 2005).

Researchers have differentiated two types of N170 tuning associated with visual recognition of print stimuli: the coarse- and fine-N170 tuning (Tong et al., 2016, Xue et al., 2023, Zhao et al., 2014), differing in the specificity or sensitivity of neural response to certain types of stimuli. Coarse-N170 tuning reflects more negative amplitudes for word or word-like stimuli compared to symbols in alphabetic scripts (Eberhard-Moscicka et al., 2015, Maurer et al., 2005, Zhao et al., 2014), as well as for valid and invalid characters compared to line drawings in logographic scripts like Chinese (Cao et al., 2011, Tong et al., 2016). Evidence shows that coarse-N170 tuning, which emerges rapidly with reading acquisition, is associated with reading skills such as fluency or accuracy (German-speaking children, Eberhard-Moscicka et al., 2015; Chinese children, Tong et al., 2016). On the other hand, the fine-N170 tuning indices a more nuanced response to the difference between words (real or pseudo) and consonant strings in alphabetical scripts (Eberhard-Moscicka et al., 2015, Zhao et al., 2014), as well as among real, pseudo, and false characters in Chinese (Tong et al., 2016, Xue et al., 2023), and tends to develop with greater accumulation of reading experience. However, the age at which fine N170 tuning emerges remains controversial: some studies have found this effect in children aged 7–9, including in German (Zhao et al., 2014) and Chinese (Tong et al., 2016, Xue et al., 2023), while others have failed to find such effects in children aged 8–11 (English, Coch and Meade, 2016). Thus, the developmental trajectory of fine N170 tuning and its link to reading outcomes in children remains under investigation.

Compared with findings on the N170 in reading tasks, little is understood about the relationship between rs-EEG and reading development in children. Rs-EEG is recorded when individuals are not required to perform any particular tasks with their eyes either closed or fixated on a point. It has been shown human brain remains active even when it is not engaged in any cognitive activities (Raichle and Mintun, 2006). This spontaneous neural activity was thought to reflect the fundamental neurobiological characteristics and dynamic intrinsic functional organizations (e.g., Deco et al., 2011, Deco et al., 2013; Sadaghiani, 2010). In addition, these activities undergo changes with aging, mirroring neural reorganization and cognitive development (Anderson and Perone, 2018).

By converting EEG signals from the time domain to the frequency domain utilizing Fourier transformation, different frequency bands, including delta (1–3 Hz), theta (4–7 Hz), alpha (8–12 Hz), beta (13–30 Hz), and gamma (30–100 Hz), can be derived. Some researchers have demonstrated that reading performance correlates with these different brain rhythms. For example, a negative correlation was found between the gamma band power and reading skills in 14- or 15-year-old English speaking teenagers (Tierney et al., 2014). Beyond that, these measures are potentially capable of predicting development of reading skills and cognitive performance in subsequent years for young children (Canadian children, Kwok et al., 2019; Chinese children: Lui et al., 2021; Meng et al., 2021; Dutch children, Schiavone et al., 2014; American children, Whedon et al., 2020). For example, Meng et al. (2021) found that the theta band power could predict vocabulary knowledge in later years by tracking the change of rs-EEG power in 7- to-11-year-old Chinese children. However, the role of resting-state EEG in the reading development of Chinese children–particularly its possible links to Chinese reading-related skills such as orthographic awareness–has yet to be systematically examined.

In contrast to alphabetic writing systems such as German and English, which feature systematic grapheme-to-phoneme correspondence, Chinese employs a logographic script characterized by deep orthography and limited sub-lexical phonological transparency (DeFrancis, 1989, McBride, 2016, Zhang et al., 2024). The basic units of Chinese writing are characters, over 80 % of which are compound characters composed of semantic and phonetic radicals (DeFrancis, 1989, Shu et al., 2003). While semantic radicals provide partial cues to meaning, phonetic radicals offer probabilistic and often inconsistent guidance on pronunciation—only about 26 % of phonograms show high phonological similarity to their phonetic radicals (Fan et al., 1984). This inconsistency poses unique challenges for beginning readers, who must extract orthographic regularities from complex visual characters without the aid of reliable orthographic -semantic and orthographic -phonological mappings. Through increasing exposure to Chinese characters, children gradually and implicitly develop orthographic awareness—an understanding of positional, structural, and functional rules governing character formation (Tong et al., 2019, Tong and McBride, 2014). As this awareness emerges, it serves as a critical metalinguistic skill that facilitates efficient character recognition and strongly predicts reading development (Deacon et al., 2019, Li and Wu, 2015, Tong and McBride-Chang, 2010, Yeung et al., 2013).

Although neural activities are widely recognized as being strongly associated with reading abilities, most evidence comes from cross-sectional studies, which capture data at a single time point (Coch and Meade, 2016, Tong et al., 2016). This leaves a gap in understanding how neural activity, during both task-related and resting states, predicts longitudinal reading development in Chinese children. To address this gap, the current study combines rs-EEG power and event-related potentials, specifically the N170 component evoked by character recognition, to explore potential neural predictors of reading development. While the impact of neural measures and reading-related cognitive skills on reading development has often been studied independently, their combined influence remains underexplored. Given the critical role of orthographic awareness in Chinese reading development and its association with the N170, this study further investigates how neural measures influence orthographic awareness and its mediating role between neural activity and reading outcomes, aiming to clarify how both neural and cognitive factors jointly contribute to reading development in Chinese children.

The first purpose of the current study is to examine the developmental trajectory of rs-EEG power and N170 amplitude during Chinese character processing. For rs-EEG power, previous studies have reported inconsistent findings regarding the relationship between any specific frequency bands and literacy skills (Kwok et al., 2019, Meng et al., 2021). This uncertainty is exacerbated by the limited research on logographic writing systems such as Chinese. Therefore, we adopted an exploratory approach to analyze major frequency bands—delta (1–3 Hz), theta (4–7 Hz), alpha (8–12 Hz), and beta (13–30 Hz)—as previous research suggests that these bands change with age and are related to reading skills (Clarke et al., 2001, Meng et al., 2021, Tierney et al., 2014). For task-state EEG, a rapid stream stimulation paradigm was adopted to present stimuli (Lochy et al., 2016, Rudell and Hua, 1997), which facilitates automatic discrimination and shortens measurement time (Walle de Ghelcke et al., 2021). To investigate the development of two types of N170 tuning, three types of Chinese characters—real, pseudo, and false—were included. Real and pseudo characters adhered to orthographic patterns, but pseudo characters lack semantic validity, while false characters violated orthographic rules. We hypothesized that both real and pseudo characters would elicit larger N170 amplitudes than false characters, reflecting the development of fine N170 tuning in children with backgrounds in both alphabetic orthographies and Chinese. In particular, we expected to observe N170 amplitude differences between real and pseudo-characters—a pattern that has been inconsistently reported in previous studies with Chinese children (Tong et al., 2016, Zhao et al., 2019).

The second purpose is to investigate the predictive effect of rs-EEG power and the amplitude of the N170 on children’s orthographic awareness and reading ability. For this purpose, we tested children’s orthographic awareness and reading performance. Correlational analyses were conducted to explore the relationship between neural activity, orthographic awareness and reading performance at each testing period. Besides, based on correlational results, structural equation model (SEM) was performed to examine the predictive effects of neural activity on orthographic awareness and reading ability. As shown in previous studies, the rs-EEG power and word-related N170 closely correlate with metalinguistic skills and reading ability in school-age children (Bach et al., 2013, Coch and Meade, 2016, Kwok et al., 2019, Whedon et al., 2020), but the causal relationship among them is uncertain. With a longitudinal experimental design, the causal relationship between variables can be revealed by controlling the autoregressive effect of the variables. We expect that children's neural activity in the first testing phase could predict the orthographic awareness and reading ability in the second phase after controlling for developmental differences in the previous period.

Finally, mediation analysis was performed to examine whether neural activity could influence reading ability through orthographic awareness. Previous research has shown that the N170 component reflects orthographic processing during visual word recognition (Cao et al., 2011, Maurer et al., 2005, Tong et al., 2016) and that orthographic awareness significantly influences reading development in Chinese children (Li and Wu, 2015, Yang et al., 2019). These findings raise the possibility that orthographic awareness may serve as a mediating cognitive mechanism. This assumption is particularly relevant in the context of Chinese reading, where orthographic awareness is expected to play a critical role in character recognition—unlike in alphabetic languages, where phonological awareness is typically more important (Ehri et al., 2001). Regarding rs-EEG power, prior studies have also found that rs-EEG affects cognitive functions through mediator variables (Broomell et al., 2021, Meng et al., 2021). Based on these considerations, we examined whether orthographic awareness would modulate the influence of rs-EEG power and N170 on children’s reading performance by constructing a hypothesized model with brain activity as the independent variable, reading outcomes as the dependent variable, and orthographic awareness as the mediator.

## 2 Methods

### 2.1 Participants

Seventy-three native Chinese children from Grade 2–6 were recruited for the present longitudinal study with one-year interval. All children were right-handed and had normal or corrected normal vision. They reported no mental or behavioral disorders, such as ADHD, autism, or any neurodevelopmental disorders. Written consents were collected from children’s parents prior to the study. This study was approved by the ethics committee of the State Key Laboratory of Cognitive Neuroscience and Learning at Beijing Normal University. To reduce sample attrition, we provided feedback to participants and their parents on task performance, enhancing their involvement and motivation. We also proactively addressed potential barriers to continued participation, such as parents' concerns about the safety of the EEG equipment.

The selected Grade range was based on the developmental trajectory of reading abilities in Chinese children, particularly during the period when reading skills and neural specialization in reading undergo significant changes (Huo et al., 2025, Meng et al., 2021, Zhang et al., 2024, Zhao et al., 2019). In Mainland China, formal reading instruction begins from Grade 1 (around age 6–7), focusing on character recognition, reading, and the use of Pinyin, a phonetic system designed to support character pronunciation. Instruction starts with simple, high-frequency characters and gradually progresses to more complex, lower-frequency characters and words as reading abilities develop with grades. As indicated by some corpus-based studies of primary school textbooks (e.g., Zhang et al., 2024), character recognition accuracy reaches stability around Grades 3–4 (9–10 years old), increasing from 85 % to 97 % by Grades 5–6. During this period, reading speed also improves, and by 5–6 graders show similar word recognition reaction times approximate adult (college students) levels, suggesting a near-automatization of decoding processes.

In the first test session (Time 1, *N* = 73), the data of 5 children in the task state EEG analysis and the data of 4 children in the rs-EEG analysis were excluded due to excessive artifacts. As a result, our analyses for Time 1 were based on the data from 68 children (34 boys, mean age = 10.4 years, *SD* = 1.6, ranging from 7.4 to 13.1 years) in the task-state condition and 69 children (35 boys, mean age = 10.4 years, *SD* = 1.6, ranging from 7.4 to 13.1 years) in the resting-state condition. Seven children dropped out of the second test session (Time 2, *N* = 66), the data of 2 children in the task state EEG analysis and the data of 1 child in the rs-EEG analysis were excluded due to data quality. Resulting in 64 children (29 boys, mean age = 11.4 years, *SD* = 1.5, ranging from 8.4 to 14.1 years) in the task-state condition and 65 children (30 boys, mean age = 11.5 years, *SD* = 1.6, ranging from 8.4 to 14.1 years) in the resting-state condition. The cross-time analysis was performed on participants who participated in both sessions.

### 2.2 Behavioral tasks

*Non-verbal intelligence Test.* Raven's Standard Progressive Matrices (Zhang and Wang, 1989) were used to assess children’s non-verbal intelligence. In this task, children were instructed to select the correct piece to complete the visual pattern from a set of 6–8 options. A total of 60 items were included. The number of correct answers was taken as the final score.

*Orthographic awareness test.* A non-character cross-out task, in which children need to cross out all non-characters as fast and accurately as possible within 40 s, was used to measure children’s orthographic awareness (Liu et al., 2017, Zou et al., 2019). Non-characters were constructed in two ways: by swapping the positions of components (e.g., “”) in real characters (e.g., “忆”) or by combining two unrelated components (e.g., “”). The test included a practice phase and a formal testing phase. The practice phase consisted of 8 non-characters and 20 real characters to familiarize children with the task rules. In the formal phase, 50 non-characters were embedded among 104 real characters, and children were asked to cross out as many non-characters as possible within a 40-second time limit. All non-characters and real characters (an 11 ×14 matrix) were presented in random order on A4 paper. The number of non-characters that are correctly identified and crossed out was calculated as final scores, with a maximum possible score of 50.

*Written Vocabulary (character) Size.* A Chinese character recognition task (Pan et al., 2016) with 150 unique print characters was used to probe children’s written vocabulary size. Children were instructed to read the characters out aloud one by one. As the test progresses, characters become more complicated and less frequent. The task terminates if 15 consecutive unknown or incorrect responses were detected. The number of correct responses was taken as the final score.

*Word List Reading.* Children’s word knowledge was assessed through a word list reading task (Zhang et al., 2012). In this task, they read 180 frequent disyllabic words as fast and accurately as possible. The number of correct responses divided by the time spent on this task was taken as the final score.

*Reading Fluency.* One hundred sentences, arranged in the order of increasing sentence length, were used to test children’s reading fluency (Su et al., 2017). Each child was given 3 min to read those sentences silently and then determine their semantic plausibility. There were 3 practice sentences and 100 experimental sentences. For example, a false response should be given to a sentence like ‘the sun rises from the west’. We calculated the difference between the number of characters in the sentences that were answered correctly and the number of characters in sentences that were answered incorrect. And the final score is this character difference divided by the time taken (3 min).

### 2.3 ERP experiment

The ERP experiment employed a Chinese character recognition task, analogous to a lexical decision task. The task included three categories of characters (real, pseudo, and false), target characters, and background stimuli (Fragment) (see Table 1). The real characters were selected from the first-grade Chinese textbook (2016 edition) with an average frequency of 768 per million (Cai and Brysbaert, 2010). Pseudo characters were constructed by combining two components from real characters while adhering to Chinese orthographic rules (e.g., the pseudo character illustrated in Table 1 consists of the left component of the real character 灯 dēng [lamp] and the right component of 奶nǎi [grandmother]). False characters, by contrast, were generated by swapping the positions of components from real characters, thereby violating orthographic rules. Each character categories containing 46 items, with 6 items from each category used as practice trials and 40 items used in the experimental trials. Both pseudo-characters and false characters were constructed using components derived from the same set of 46 real characters. All three categories with left-right configurations and matched stroke counts, ranging from 5 to 10 strokes. Eight animal names, such as “猫” (cat) and “狗” (dog), were selected as target characters, all of which were familiar to all participants. Background stimuli consisted of 2107 fragments derived by dividing real characters into 16 square pieces and randomly rearranging them.

**Table 1:** Examples of four types of stimuli.

Type	Example	Stroke	Radical	Radical position	Pronounceable
Real		√	√	√	√
Pseudo		√	√	√	×
False		√	√	×	×
Fragment		×	×	×	×

**Fig. 1:**
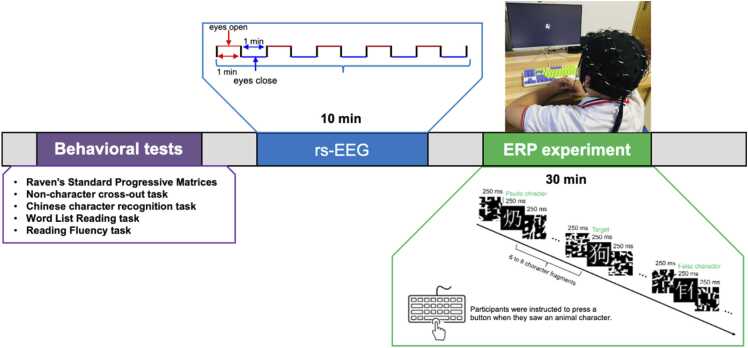
Experimental procedure. Participants were instructed to completed 3 sessions of the experiment: (i) 5 behavioral tasks; (ii) rs-EEG recording session, (iii) Chinese character recognition task.

In the Chinese character recognition task (see Fig. 1. ERP experiment), stimuli were presented using a rapid stream stimulation paradigm (Lochy et al., 2016, Peter Rudell, 1992). Each trial started with 6–8 character fragments, followed by a non-target character (real, pseudo or false) or a target character (animal name). Each stimulus was presented for 250 ms (ms) without any interval. Participants were instructed to press a button when they saw a target character. We repeated the target characters 5 times and non-target stimuli twice, yielding 40 target trials and 240 non-target trials. A target trial was often followed by 4-to-8 non-target trials. Real, pseudo and false characters in the non-target stimuli were presented in a pseudo-randomized order, to make sure that two consecutive trials were not the same type. Before the formal experiment, 21 practice trials were provided for participants to become familiar with the procedure. All trials were split into 14 blocks, with each block containing 20 trials (2–3 are target trials). Different Blocks were presented in a randomized order. All stimuli were presented using E-prime 3.0 software (Psychology Software Tools, Inc) on a 21-inch monitor with a 60 Hz refresh rate. Participants took a short break between each block. The whole ERP experiment last for approximately 30 min including breaks between blocks.

### 2.4 Procedure

For two test sessions (T1 and T2), all children completed a battery of behavioral tasks: with one task related to IQ testing, one evaluating orthographic awareness, and three measuring reading abilities. All tasks were administered individually, except for the IQ test, which was conducted in groups of 10–15 children. Subsequently, children underwent a continuous rs-EEG recording session, followed ERP data collection during the Chinese character recognition task (see Fig. 1).

The rs-EEG recording session consisted of a 10-minute protocol alternating between 1-minute eyes-open and 1-minute eyes-closed conditions, repeated five times. During the recording, children sat in front of a computer and keep their head and body still. Upon hearing ‘Please open y*our eyes’*, they fixed on a fixation cross “+ ” at the center of the screen. Upon hearing “Please close your eyes,” they closed their eyes while remaining awake. For subsequent analyses, only the data from the eyes-open condition were used. Prior research has suggested that resting-state EEG recorded during eyes-open conditions may be more suitable for revealing associations between neural activity and individual differences in cognitive performance, such as academic achievement (Cheung et al., 2014).

### 2.5 EEG recording and preprocess

The EEG data were recorded with a portable NeuroScan SynAmps amplifier with a 32-channel Ag/AgCl cap, following the according to the international 10–20 system. The right mastoid served as the online reference, and an electrode between FP1 and FF2 served as the ground. Horizontal electrooculograms (HEOG) was recorded from an electrode placed beside the canthus of the right eye, while vertical electrooculograms (VEOG) were recorded an electrode positioned below the left eye. All channels were recorded at a sampling rate of 1000 Hz with a band pass filter of 0.1–100 Hz, and electrode impedance were maintained below 5 kΩ.

Offline preprocessing of EEG data was conducted using EEGLAB v2021.1 (Delorme and Makeig, 2004) in MATLAB. First, data were re-referenced to the average reference, resampled at 500 Hz, and digitally band-pass filtered (0.1–30 Hz). Then, we ran an independent component analysis (ICA) to identify and remove artifact-related components. Components associated with eye blinks, eye movements, head motion, and muscle activity were manually excluded based on their frequency spectra and scalp topographies (Hu and Zhang, 2019). The mean (SD; range) number of components rejected were as follows: 3.82 (1.21; 2–5), 4.08 (1.34; 2–8) for rs-EEG at Time 1 and Time 2; 4.11 (1.18; 2–6), 3.98 (1.09; 2–6) for task state EEG at Time 1 and Time 2. The above preprocessing steps were applied consistently to both resting state and task state EEG data across all participants.

For rs-EEG, data of 2 s before and after every auditory instruction was removed. Then, the continuous EEG data were subsequently down sampled to 250 Hz and segmented into 2-second epochs with 1-second overlap. Epochs with voltage changes exceeding ±100 µV in any channel were rejected. On average, the number of retained epochs was 260.26 (SD = 28.71; range = 85–271) for Time 1 and 255.29 (SD = 15.38; range =167–268) for Time 2. Using the Fast Fourier Transform (FFT) in MATLAB (Frigo and Johnson, 1998), the signal of each epoch (epoch size = 2000 ms, overlap = 50 %, and frequency resolution = 0.5 Hz) was transformed onto the frequency domain. EEG power in delta, theta, alpha, and beta frequency bands was extracted, and the average power of each band across electrodes in the temporo-parietal and occipital regions (T3, T4, T5, T6, C3, CZ, C4, CP3, CPZ, CP4, P3, PZ, P4, O1, OZ) was calculated (Brem et al., 2006, Li et al., 2013).

For the task-state EEG, data were segmented from −100–600 ms relative to the stimulus onset and baseline-corrected using the pre-stimulus interval (-100–0 ms). Epochs with voltage changes exceeding ±100 µV in any channel were rejected. After rejection, the average number of retained trials per condition was as follows: Time 1 – Real characters: 73.61 (SD = 4.23; range = 59–79), Pseudo-characters: 73.99 (SD = 4.05; range = 60–78), False characters: 73.27 (SD = 4.87; range = 58–78); Time 2 – Real characters: 72.80 (SD = 3.69; range = 61–77), Pseudo-characters: 72.30 (SD = 3.17; range = 60–76), False characters: 72.80 (SD = 3.43; range = 63–78). Based on prior research on word-related N170 in children and adults (e.g., Li et al., 2013; Maurer et al., 2006; Zhao et al., 2018), channels over the occipito-temporal regions with the maximum negative amplitude (T5/T6) were selected for analysis (Location of T5/T6 see Fig. 6B). Data within a ± 30 ms time window around the latency of the average peak amplitude (determined by averaging the data across all participants to identify the peak latency (180–400 ms)) was extracted for statistical analysis.

### 2.6 Data analyses

Resting EEG power was analyzed using a 2 (Time: Time 1, Time 2) × 4 (Band: delta, theta, alpha, beta) ANOVA, and N170 mean amplitudes were analyzed with a 2 (Time: Time 1, Time 2) × 3 (Stimulus type: real, pseudo, false character) × 2 (Electrodes: T5, T6) ANOVA. A correction according to Greenhouse and Geisser (1959) was applied for sphericity violations, and Bonferroni correction was used for multiple comparisons. All reported results reflect these adjustments. Pearson correlation analysis examined the relationships between neural activity (rs-EEG power and N170 amplitudes at Time 1 and Time 2), orthographic awareness, and reading abilities (character recognition, word reading, reading fluency).

**Fig. 2:**
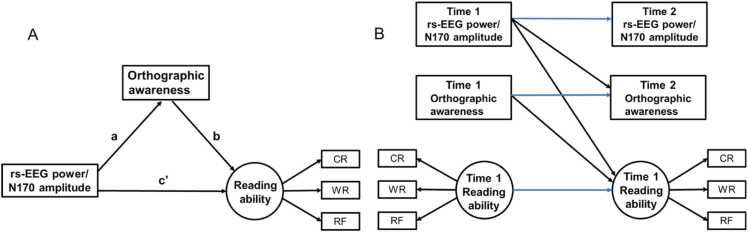
Hypothesized structural equation models. (A)The hypothetical model for the cross-sectional data of Time 1 and Time 2. (B)The hypothesis model based on longitudinal data. a: effects of rs-EEG power/N170 amplitude on orthographic consciousness; b: effect of orthographic awareness on reading ability; c ': direct effect of rs-EEG power/N170 amplitude on reading ability after controlling for the mediating effect of orthographic awareness. The indirect effect of rs-EEG power/N170 amplitude on reading ability through orthographic awareness can be expressed as: a × b. CR: character recognition; W*R*: word reading; RF: reading fluency.

Confirmatory Factor Analysis (CFA) and Structural Equation Modeling (SEM) addressed three key questions: 1) Do neural activities influence orthographic awareness and reading performance at both Time 1 and Time 2? 2) Is the effect of neural activity on reading mediated by orthographic awareness? 3) Can neural activity at Time 1 predict orthographic awareness and reading performance at Time 2? Hypothesized structural equation models (Fig. 2) were constructed to test these questions. CFA was used to extract two latent factors: reading ability (character recognition, word reading, reading fluency) and N170 amplitude (mean amplitudes for false, pseudo, and real characters).

SEM, performed using maximum likelihood estimation in Mplus 8.3, examined direct and indirect effects. Mediation analysis utilized the Bootstrap method with 5000 resamples and 95 % confidence intervals (CIs). Model fit was evaluated using the following criteria: χ^2^/df should be within the range of 1–3 with *p* > .05 (Bentler and Bonett, 1980, Hair, 2010); Root Mean Square Error of Approximation (RMSEA) < .08, Standardized Root Mean Square Residual (SRMSR) < .08, Comparative Fit Index (CFI) and Tucker-Lewis Index (TLI) > .90 (Bentler and Bonett, 1980, Hu and Bentler, 1999). SEM analyses were conducted with and without controlling for age. In addition, to address potential developmental moderation effects, we further examined whether age moderated the effect of neural activity (i.e., rs-EEG power and N170 amplitude) on orthographic awareness and reading ability, as well as the indirect pathway from neural activity to reading via orthographic awareness. Moderated mediation models were tested by incorporating interaction terms between neural indicators and age into the structural models. These moderation analyses were conducted only on the cross-sectional datasets (Time 1 and Time 2, respectively) because incorporating age moderation into longitudinal models led to non-convergence due to increased complexity and potential overparameterization. All the reported SEM results in the current study are standardized.

## 3 Results

### 3.1 Behavioral tasks

Results of four behavioral tests conducted over two consecutive years are shown in Table 2. Paired *t-*tests indicated enhanced performance at Time 2 compared to Time 1 across all tests (*ps* ≤ 0.004). According to the norms provided by H.-C. Zhang and Wang (1989), the non-verbal intelligence development of the children in the present study was within the normal range.

**Table 2:** Children’s behavioral tests performance.

	Time 1				Time 2				*t* (59)	*p*	Cohen's *d*
	N	Min	Max	M (SD)	N	Min	Max	M (SD)			
Orthographic awareness	69	17	49	35.43 (8.79)	66	23	50	39.18 (7.33)	−5.085	< 0.001	−0.657
Character recognition	69	27	133	102.08 (22.95)	66	29	134	114.89 (18.05)	−8.782	< 0.001	−1.134
Word reading	69	5.58	135.20	86.20 (20.78)	66	44.04	149.35	92.80 (18.89)	−2.992	< 0.001	−0.386
Reading fluency	69	33.92	519.00	209.79 (93.94)	66	61.67	570.67	283.97 (112.15)	−6.122	< 0.001	−0.790

### 3.2 Resting-state EEG

**Fig. 3:**
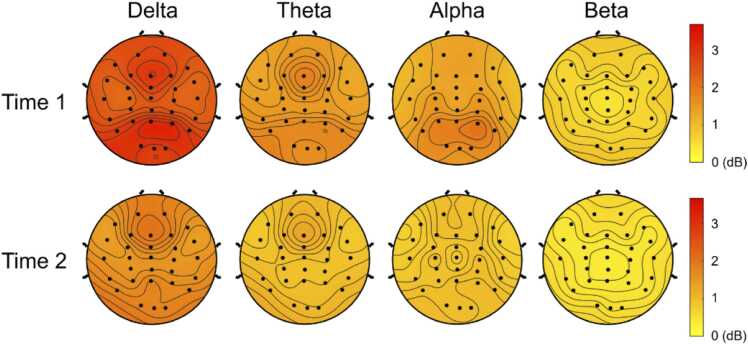
Topographic maps for delta, theta, alpha and beta band EEG power at Time 1 and Time 2.

Fig. 3 illustrates topographic maps of delta, theta, alpha and beta band EEG power at Time 1 and Time 2. Results of ANOVAs showed that there was a significant main effect of testing Time, *F*(1,58) = 155.75, *p* < 0.001, suggesting that the EEG power was significantly weaker in the Time 2 (1.02 ±.03) than the Time 1 (1.61 ±.04). There was also a main effect of power Band, *F*(3174) = 619.91, *p* < 0.001, indicating that delta band EEG power (2.17 ±.50) was significantly larger than theta (1.28 ±.34), alpha (1.26 ±.46) and beta band (.54 ±.12). Additionally, theta band EEG power was significantly larger than beta band but not alpha band, and alpha band was significantly larger than beta band. The interaction between testing Time and power Band was also significant, *F*(3174) = 85.20, *p* < 0.001. Further simple effect tests revealed that the EEG power for all 4 bands decreased in Time 2 significantly, including delta (*F*(1,58) = 154.20, *p* < 0.001), theta (*F*(1,58) = 161.08, *p* < .001), alpha (*F*(1,58) = 97.64, *p* < .001), beta (*F*(1,58) = 118.94, *p* < .001).

The correlation matrix among the averaged EEG power of the four bands, orthographic awareness, and reading performance is shown in Table 3. Firstly, at Time 1, EEG power of delta and theta bands negatively correlated with the orthographic awareness, character recognition, and word reading. Secondly, at Time 2, EEG power of delta, theta and alpha bands negatively correlated with the orthographic awareness; theta band EEG power negatively correlated with reading fluency; beta band EEG power negatively correlated with word reading. Finally, negative correlations were found between the delta band in Time 1 and the orthographic awareness, character recognition, word reading, and reading fluency in Time 2, as well as the theta band in Time 1 and the orthographic awareness, character recognition, and reading fluency.

**Table 3:** Correlation matrix among averaged rest EEG power of the four bands, orthographic awareness, and reading performance (uncorrected for multiple comparisons).

	1	2	3	4	5	6	7	8	9	10	11	12	13	14	15	16
1.T1 OA	1															
2.T1 CR	.587**	1														
3.T1 W*R*	.474**	.731**	1													
4.T1 RF	.527**	.592**	.508**	1												
5.T1 delta	−.314**	−.395**	−.258*	−.268*	1											
6.T1 theta	−.351**	−.369**	−0.234	−.267*	.877**	1										
7.T1 alpha	−0.072	−0.018	0.012	−0.06	.640**	.580**	1									
8.T1 beta	−0.12	−0.045	−0.08	0.063	.542**	.474**	.497**	1								
9.T2 OR	.643**	.525**	.332**	.431**	−.362**	−.397**	−0.173	−0.078	1							
10.T2 CR	.481**	.857**	.741**	.634**	−.401**	−.266*	−0.091	−0.132	.375**	1						
11.T2 W*R*	.314*	.672**	.678**	.465**	−.318*	−0.212	−0.044	−0.111	.413**	.668**	1					
12.T2 RF	.376**	.689**	.642**	.673**	−.340**	−.283*	−0.134	−0.051	.428**	.641**	.676**	1				
13.T2 delta	−.291*	−0.149	−0.113	−0.2	0.247	.318*	−0.012	0.125	−.324**	−0.168	−0.225	−0.199	1			
14.T2 theta	−.299*	−0.148	−0.132	−.269*	0.216	.415**	−0.007	0.113	−.328**	−0.153	−0.185	−.250*	.942**	1		
15.T2 alpha	−.278*	−0.102	−0.092	−0.148	0.242	.346**	.374**	.310*	−.316**	−0.118	−0.123	−0.16	.672**	.701**	1	
16.T2 beta	−0.129	−0.147	−0.116	−0.206	0.137	0.171	0.095	.322*	−0.123	−0.201	−.257*	−0.16	.657**	.626**	.651**	1

*Note*. T1: time 1; T2: time 2;OR: orthographic awareness; CR: character recognition; W*R*: word reading; RF: reading fluency; Delta, mean power of delta band; Theta, mean power of theta band; Alpha, mean power of alpha band; Beta, mean power of beta band. ** *p* < .01, * *p* < .05.

Based on the correlation results, we mainly focused on the cross-sectional and longitudinal effects of delta and theta band EEG power on orthographic awareness and reading performance (character recognition, word reading and reading fluency). Moreover, we examined the mediation mechanisms from delta and theta band EEG power via orthographic awareness to reading performance.

#### 3.2.1 Cross-sectional effects of delta and theta band EEG power at T1 and T2

The SEM analyses conducted at T1 and T2 to examine the effects of Delta and Theta band EEG power on reading ability, as well as the mediating role of orthographic awareness. Model fit indices for all models were within acceptable ranges, detailed results were reported below. Further analysis showed that controlling for age reduced model fit at T1 but not at T2. As the predictive effects of theta and delta power on orthographic awareness and reading ability were non-significant, detailed results are not reported. The moderated mediation model, in which age was specified as a moderator, also demonstrated acceptable fit, significant findings are reported below. Detailed model fit indices are presented in Table 5.

**Fig. 4:**
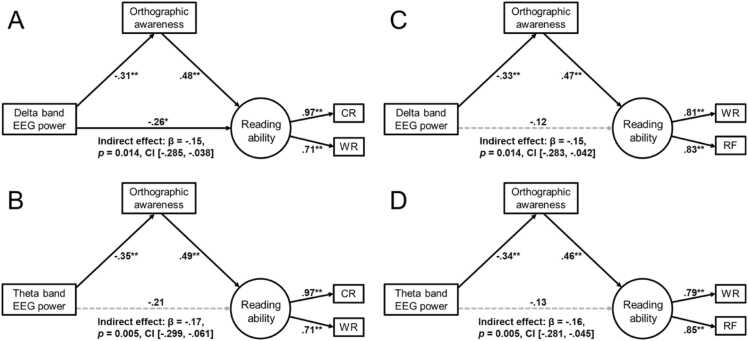
Standardized parameter estimates of the direct and indirect associations of the rs-EEG power with the reading performance at Time 1 and Time 2. The effects of delta (**A**) and theta (**B**) band EEG power on orthographic awareness and reading performance at Time 1. The effects of delta (**C**) and theta (D) band EEG power on orthographic awareness and reading performance at Time 2. CR: character recognition; W*R*: word reading; RF: reading fluency. ** *p* < .01, * *p* < .05.

At T1 (Fig. 4A), Delta band EEG power significantly negatively predicted orthographic awareness (β = - 0.31, *p* = 0.005), and orthographic awareness further significantly positively predicted reading ability (β = 0.48, *p* < 0.001). Meanwhile, the direct effect of Delta band EEG power on reading ability was also significant (β = −0.26, *p* = 0.019), and the indirect effect via orthographic awareness was significant (β = −0.15, *p* = 0.014, 95 % CI = [-0.29, −0.04]). The latent factor (reading ability) extracted based on character recognition and word reading had relatively good loading coefficients, βs > 0.71 for both paths. In addition, moderated mediation model showed that the interaction between Delta power and age significantly predicted reading ability (β = 1.19, *p* = 0.022, 95 % CI = [0.534, 2.611], suggesting that age moderated the direct effect of Delta power on reading. Specifically, this negative effect was significant among younger children (8.6 years; b = −11.02, *p* = .036, 95 % CI = [-21.31, −0.73]), but diminished with increasing age and was no longer significant at the mean (10.6 years) or higher (12.1 years) age levels (*ps* >.174).

At T2 (Fig. 4B), Delta band EEG power still significantly negatively predicted orthographic awareness (β = −0.33, *p* = 0.002), and orthographic awareness significantly positively predicted reading ability (β = 0.47, *p* < 0.001). However, the direct effect on reading ability was not significant (β = −0.12, *p* = 0.336), but the indirect effect via orthographic awareness remained significant (β = −0.16, *p* = 0.014, 95 % CI = [-0.28, −0.04]). The latent factor had relatively good loading coefficients, βs > 0.71 for both paths (character recognition and word reading) within the reading ability. Notably, age significantly moderated the effect of Delta band power on orthographic awareness, as indicated by a significant interaction term (Delta × Age) (β = 2.87, *p* = 0.045, 95 % CI [0.04, 3.60]), Delta power negatively predicted orthographic awareness (b = −8.56, *p* = .020, 95 % CI [−15.72, −1.40]) at a younger age (9.6 years), while this effect was not significant at average (11.6 years) or older (13 years) ages (*ps* > 0.133). A similar age-dependent pattern emerged for the indirect effect, Delta band power significantly predicted reading ability via orthographic awareness (b = −0.42, BootSE = 0.28, 95 % CI [−1.13, −0.03]) at a younger age (9.6 years), while this effect was not significant at average (11.6 years) or older (13.1 years) ages, as the 95 %CI included zero.

At both T1 (Fig. 4C) and T2 (Fig. 4D), Theta band EEG power significantly negatively predicted orthographic awareness (T1: β = −0.35, *p* = 0.001; T2: β = −0.34, *p* = 0.001), and orthographic awareness also significantly positively predicted reading ability (T1: β = 0.49, *p* < 0.001; T2: β = 0.46, *p* < 0.001). However, the direct effect of Theta band EEG power on reading ability was not significant at either time point (T1: β = −0.21, *p* = 0.097; T2: β = −0.13, *p* = 0.294), but the indirect effect via orthographic awareness was significant (T1: β = −0.17, *p* = 0.005, 95 % CI = [-0.30, −0.06]; T2: β = −0.16, *p* = 0.011, 95 % CI = [-0.28, −0.05]). The latent factor had relatively good loading coefficients (T1: βs > 0.81; T2: βs > 0.79) for both paths (word reading and reading fluency) within the reading ability. The moderated mediation model showed that the interaction between theta and age significantly predicted reading ability (β = 1.40, *p* = .002, 95 % CI [0.54, 2.32]) at T1, indicating age-dependent moderation. Specifically, theta power negatively predicted reading ability at younger ages (8.6 years; b = −16.90, *p* = .047, 95 % CI [−33.55, −0.24]), was not significant at the average age (10.6 years; p = .525), and reversed to a significant positive effect at older ages (12.1 years; b = 21.01, *p* = .049, 95 % CI [0.09, 41.93]). While at T2, the moderating effect of age was only observed on the indirect pathway: theta power significantly predicted reading ability via orthographic awareness at younger ages (9.6 years; b = −0.59, BootSE = 0.37, 95 % CI = [-1.46, −0.04]), but not at average (11.6 years) or older ages (13.1 years), as the 95 %CI included zero.

#### 3.2.2 Longitudinal effects of delta and theta band EEG power across two time periods

**Fig. 5:**
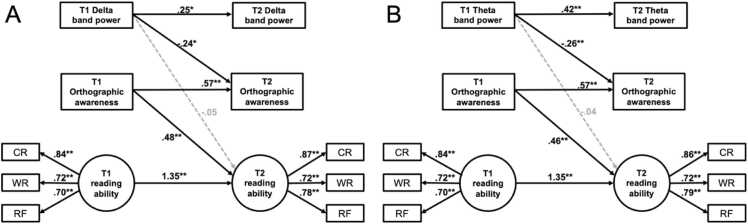
Standardized parameter estimates of the direct and indirect associations of the delta (A) and theta (B) band rs-EEG power with the reading performance across time. Horizontal arrow indicates the autoregressive effect. T1: Time 1; T2：Time 2; CR: character recognition; W*R*: word reading; RF: reading fluency. ** *p* < .01, * *p* < .05.

The longitudinal SEM results for delta band EEG power predicting orthographic awareness and reading performance are shown in Fig. 5A. The model fit was acceptable, with χ^2^(27) = 33.138, *p* = 0.193, RMSEA = 0.062, SRMR = 0.063, CFI = 0.979, and TLI = 0.966. The model explained 25.7 % of the variance in reading ability at T2. After controlling for autoregressive effects of variables, the delta band EEG power at T1 significantly predicted orthographic awareness at T2 (β = −0.24, *p* = 0.013), but it did not predict reading ability (β = −0.05, *p* = 0.593); Orthographic awareness at T1 significantly predicted reading ability at T2 (β = 0.48, *p* = 0.001). However, when age (T1) was included as a control variable, the model fit deteriorated (χ^2^(35) = 113.155, *p* < 0.001, RMSEA = 0.195, SRMR = 0.244, CFI = 0.755, TLI = 0.637), and only orthographic awareness at T1 significantly predicted reading ability at T2 (β = 0.26, *p* = 0.042).

For theta band EEG power, the longitudinal SEM results are shown in Fig. 5B. The model fit was also acceptable, with χ^2^(27) = 34.587, *p* = 0.150, RMSEA = 0.069, SRMR = 0.063, CFI = 0.974, and TLI = 0.958. The model explained 28.3 % of the variance in reading ability at T2. Theta band EEG power at T1 significantly predicted orthographic awareness (β = −0.256, *p* = 0.007) but not reading ability (β = −0.039, *p* = 0.628). Orthographic awareness at T1 significantly predicted reading ability at T2 (β = 0.46, *p* = 0.001). Including age (T1) as a control variable resulted in poor fit (χ2(35) = 114.318, *p* < 0.001, RMSEA = 0.196, SRMR = 0.215, CFI = 0.752, TLI = 0.631), and only orthographic awareness at T1 significantly predicted reading ability at T2 (β = 0.31, *p* = 0.027).

In summary, both delta and theta band EEG power showed no direct effects on reading ability (except for delta band EEG power at T1). However, both EEG bands significantly influenced reading ability through orthographic awareness cross-sectionally. Moreover, delta and theta band EEG power at T1 could also predict the orthographic awareness at T2 but not reading performance. More importantly, the moderated mediation model revealed the dynamic role of age in modulating the effects of EEG power on orthographic awareness and reading performance. Both Delta and Theta power showed age-dependent effects on reading ability and orthographic awareness, with younger children being more influenced by these neural markers, while the effects diminished or reversed as age increased.

### 3.3 Task-state EEG

**Fig. 6:**
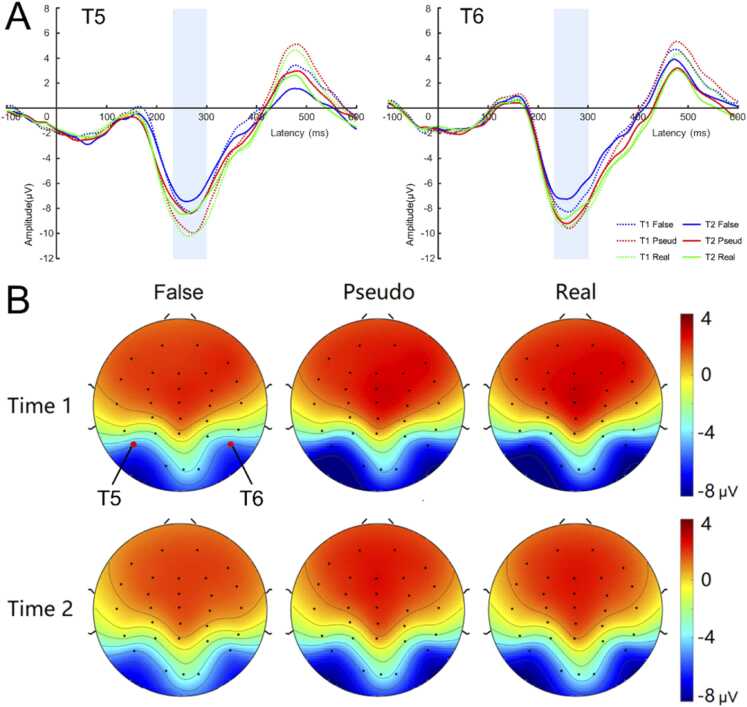
Grand average N170 waveform (A) and Topographic maps (B). T1: Time 1; T2: Time 2; Real: real character; False: false character; Pseudo: pseudo character.

The grand average waveform and topographic maps for false, pseudo and real characters over T5/T6 electrodes at both Time 1 and Time 2 are shown in Fig. 6. All three conditions elicited a clear N170 around 280 ms over occipito-parietal region at both Time 1 and Time 2. There was no obvious difference between the left and right hemispheres. Note that in the current experimental paradigm, stimuli were presented consecutively, resulting in a delayed N170 latency compared to paradigms with longer inter-stimulus intervals (ISI) (Luo et al., 2021). Besides, the baseline irregularity of the waveform may be due to the periodic presentation of background stimuli.

ANOVA results revealed a significant main effect of Stimulus type, *F*(2, 118) = 58.72, *p* < 0.001, *η*_*p*_^*2*^ = 0.50. N170 amplitudes for real characters (-7.28 ± 0.37 μV) and pseudo characters (-7.19 ± 0.37 μV) were more negative than those for false characters (-5.97 ± 0.32 μV), *ps* < 0.001. There was no significant difference between real and pseudo characters (*p* = 1.000). A significant three-way interaction between Time, Stimulus type, and Hemisphere was found, *F*(2118) = 6.69, *p* = 0.002, *η*_*p*_^*2*^ = 0.10. We further performed a 3 (Stimulus types) by 2 (Hemisphere) ANOVA analysis for two time points, respectively.

For Time 1, the main effect of Stimulus type was significant, *F*(2, 134) = 37.48, *p* < 0.001, *η*_*p*_^*2*^ = 0.36. N170 amplitudes for real characters (-7.66 ± 0.44 μV) and pseudo characters (-7.47 ± 0.45 μV) were more negative than that for false characters (-6.26 ± 0.39 μV), *ps* < 0.001, with no significant difference between real and pseudo characters (*p* = 0.307). The main effect of the Hemisphere and the interaction between Stimulus type and Hemisphere were not significant (*ps* ≥ 0.082).

For Time 2, the main effect of Stimulus type was significant, *F* (2, 130) = 30.61, *p* = 0.016, *η*_*p*_^*2*^ = 0.32, with more negative N170 for real characters (-6.62 ± 0.46 μV) and pseudo characters (-6.72 ± 0.46 μV) compared to false characters (-5.53 ± 0.40 μV), *p* < .001. No significant difference was found between real and pseudo characters (*p* = 0.442). The main effect of Hemisphere was not significant, *F*(1, 65) = 0.14, *p* = 0.713, *η*_*p*_^*2*^ = 0.002, but the interaction between Stimulus type and Hemisphere was significant, *F* (2, 130) = 5.83, *p* = 0.004, *η*_*p*_^*2*^ = 0.08. Simple effect analyses showed that the amplitude of N170 was comparable between electrode T5 and T6, with more negative N170 for real characters and pseudo characters relative to false characters (*ps* < 0.001), but no significant difference between real and pseudo characters (*ps* ≥ 0.105).

As shown in Table 4, at Time 1, results showed that the N170 amplitudes of real, pseudo and false characters positively correlate with the orthographic awareness. In addition, the N170 amplitude of real characters positively correlated with the character recognition. At Time 2, the N170 amplitudes of real, pseudo and false characters positively correlated with the orthographic awareness; besides, the N170 amplitude of real characters positively correlated with the word reading and reading fluency. Moreover, the amplitudes of N170 for real, pseudo and false characters tested at Time 1 correlated with the orthographic awareness and word reading score at Time 2. These results indicated that children with less negative N170 effect had better performance on behavioral tasks across time.

**Table 4:** Correlation matrix among averaged N170 amplitude of Stimulus Types, orthographic awareness, and the reading ability (uncorrected for multiple comparisons).

	1	2	3	4	5	6	7	8	9	10	11	12	13	14
1.T1 OA	1													
2.T1 CR	.552**	1												
3. T1 W*R*	.492**	.747**	1											
4. T1 RF	.449**	.498**	.438**	1										
5. T1 AF	.277*	0.225	0.211	0.066	1									
6. T1 AP	.295*	0.236	0.172	0.077	.926**	1								
7. T1 AR	.321**	.280*	0.222	0.126	.920**	.918**	1							
8. T2 OA	.616**	.500**	.355**	.415**	.315*	.341**	.409**	1						
9. T2 CR	.479**	.861**	.753**	.513**	0.146	0.146	0.147	.367**	1					
10. T2 W*R*	.328*	.702**	.667**	.414**	0.251	.274*	0.251	.456**	.697**	1				
11. T2 RF	.368**	.693**	.635**	.634**	0.007	0.038	0.029	.438**	.646**	.676**	1			
12. T2 AF	0.224	0.169	0.242	.292*	0.195	0.175	0.247	.325**	0.13	0.184	.247*	1		
13. T2 AP	0.198	0.178	0.247	.261*	.257*	0.236	.322*	.333**	0.132	0.245	0.208	.916**	1	
14. T2 AR	0.195	0.205	0.248	.321*	0.2	0.185	.288*	.324**	0.141	.267*	.262*	.894**	.951**	1

*Note*: OR: orthographic awareness; CR: character recognition; W*R*: word reading; RF: reading fluency; AF, mean amplitude of N170 evoked by false characters; AP, mean amplitude of N170 evoked by pseudo characters; AR, mean amplitude of N170 evoked by real characters. ** *p* < .01, * *p* < .05.

Based on the correlation results, we focused on the cross-sectional and longitudinal effects of the N170 amplitude on the orthographic awareness and the reading performance (character recognition, word reading and reading fluency), and whether the orthographic awareness mediates the effect of N170 amplitude on the reading ability.

#### 3.3.1 Cross-sectional effects of N170 amplitude at T1 and T2

The SEM analyses conducted at T1 and T2 to examine the effects of N170 amplitude on reading ability, as well as the mediating role of orthographic awareness. The model fit indices for all models were within acceptable levels, and detailed results are reported below. While further analysis was controlled for age (T1), showed poor model fit at both time points, and therefore, detailed path results are not reported. In addition, the model in which age was specified as a moderator demonstrated good fit, and the significant results are presented below. The model fit indices for the above analyses are shown in Table 5.

**Table 5:** Predictive Effects of EEG Power and N170 amplitude at T1 and T2.

Neural marker	Time Point	Model Fit	Path 1	Path 2	Direct Effect	Indirect Effect	R^2^	Model Fit with age as moderator	Model Fit with age controlled
Delta	T1	χ²(1)= 0.176, *p* = 0.675 RMSEA< 0.001 SRMR= 0.008 CFI= 1.000 TLI= 1.066	β= −0.31, *p* = 0.005	β= 0.48, *p* < 0.001	β= −0.26, *p* = 0.019	β= −0.15, *p* = 0.014 CI= [−0.29, −0.04]	0.372	χ²(3)= 5.241, *p* = 0.155, RMSEA= 1.104, SRMR= 0.039, CFI= 0.983, TLI= 0.933	χ²(2)= 3.434, *p* = 0.180 RMSEA= 0.102 SRMR= 0.019 CFI= 0.989 TLI= 0.949
Delta	T2	χ²(1)= 0.160, *p* = 0.689RMSEA< 0.001 SRMR= 0.007 CFI= 1.000 TLI= 1.089	β= −0.33, *p* = 0.002	β= 0.47, *p* < 0.001	β= −0.12, *p* = 0.336	β= −0.16, *p* = 0.014 CI= [−0.28, −0.04]	0.270	χ²(3) = 2.604, *p* = 0.457, RMSEA< 0.001, SRMR= 0.018, CFI= 1.000, TLI= 1.019	χ²(2)= 2.194, *p* = 0.334 RMSEA= 0.039 SRMR= 0.019 CFI= 0.998 TLI= 0.989
Theta	T1	χ²(1)= 0.272, *p* = 0.602 RMSEA< 0.001 SRMR= 0.010 CFI= 1.000 TLI= 1.059	β= −0.35, *p* = 0.001	β= 0.49, *p* < 0.001	β= −0.21, *p* = 0.097	β= −0.17, *p* = 0.005 CI= [−0.30, −0.06]	0.350	χ²(3)= 5.152, *p* = 0.161, RMSEA= 0.102, SRMR= 0.042, CFI= 0.984, TLI= 0.936	χ²(2)= 3.129, *p* = 0.209 RMSEA= 0.090 SRMR= 0.020 CFI= 0.991 TLI= 0.959
Theta	T2	χ²(1)= 0.291, *p* = 0.589 RMSEA< 0.001 SRMR= 0.010 CFI= 1.000 TLI= 1.075	β= −0.34, *p* = 0.001	β= 0.46, p < 0.001	β= −0.13, *p* = 0.294	β= −0.16, *p* = 0.011 CI= [−0.28, −0.05]	0.271	χ²(3)= 3.148, *p* = 0.369, RMSEA= 0.028, SRMR= 0.029, CFI= 0.998, TLI= 0.993	χ²(2)= 2.805, *p* = 0.246 RMSEA= 0.079 SRMR= 0.023 CFI= 0.990 TLI= 0.956
N170	T1	χ²(7)= 4.720, *p* = 0.694 RMSEA< 0.001，SRMR= 0.015，CFI= 1.000，TLI= 1.014	β= 0.29, *p* = 0.006	β= 0.57, p < 0.001	β= 0.10, *p* = 0.376	β= 0.17, *p* = 0.021 CI= [0.03, 0.31]	0.370	χ²(3)= 4.540, *p* = 0.209, RMSEA= 0.087, CFI= 0.988, TLI= 0.951, SRMR = 0.027	χ²(11)= 24.754, *p* = 0.010 RMSEA= 0.136 SRMR= 0.166 CFI= 0.966 TLI= 0.936
N170	T2	χ²(12)= 11.479, *p* = 0.488 RMSEA< 0.001 SRMR= 0.028 CFI= 1.000 TLI= 1.002	β= 0.34, *p* = 0.001	β= 0.48, *p* < 0.001	β= 0.10, *p* = 0.455	β= 0.16, *p* = 0.016, CI= [0.05, 0.32]	0.273	χ²(3)= 2.700, *p* = 0.440, RMSEA< 0.001, SRMR= 0.027, CFI= 1.000, TLI= 1.041	χ²(11)= 26.222, *p* = 0.071 RMSEA< 0.092 SRMR= 0.111 CFI= 0.977 TLI= 0.963

*Note*: Neurophysiological measures: delta/theta band EEG power, N170 amplitude.

Path 1: neurophysiological measures → Orthographic Awareness.

Path 2: Orthographic Awareness → Reading Ability.

Direct Effect: Neurophysiological measures → Reading Ability.

Indirect Effect: Neurophysiological measures → Orthographic Awareness → Reading Ability.

**Fig. 7:**
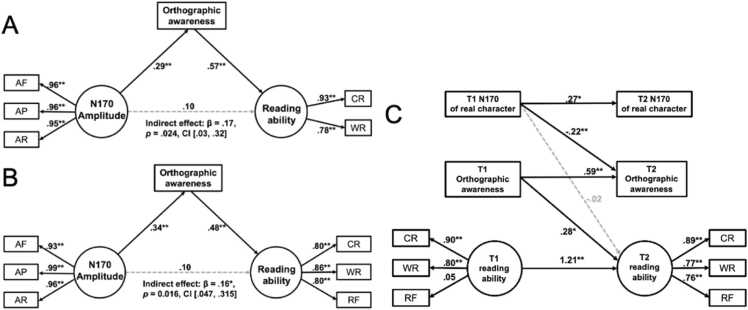
Standardized parameter estimates of the direct and indirect associations of the N170 amplitude with the reading ability. The effects of N170 amplitude on orthographic awareness and reading performance at Time 1 (A) and Time 2 (B). (**C**) The predictive effect of N170 amplitude evoked by real characters on the orthographic awareness and reading ability across time. AF: mean amplitude of N170 evoked by False characters; AP: mean amplitude of N170 evoked by Pseudo characters; AR: mean amplitude of N170 evoked by Real characters. CR: character recognition; W*R*: word reading; RF: reading fluency. ** *p* < .01, * *p* < .05.

At both T1 (Fig. 7A) and T2 (Fig. 7B), N170 amplitude significantly predicted orthographic awareness (T1: β = 0.29, *p* = 0.006; T2: β = 0.48, *p* < 0.001), and orthographic awareness significantly predicted reading ability (T1: β = 0.57, *p* < 0.001; T2: β = 0.48, *p* < 0.001). However, the direct effect of N170 amplitude on reading ability was not significant at either time point (T1: β = 0.10, *p* = 0.376; T2: β = 0.10, *p* = 0.455), but the indirect effect via orthographic awareness was significant (T1: β = 0.17, *p* = 0.021, 95 % CI = [0.03, 0.31]; T2: β = 0.16, *p* = 0.016, 95 % CI = [0.05, 0.32]). The latent factors had relatively good loading coefficients for all paths (N170 amplitude of real, pseudo and false characters) within the N170 amplitude (T1: βs > 0.95; T2: βs > 0.93), and for two paths (character recognition and word reading) within the reading ability (T1: βs > 0.78; T2: βs > 0.80).

The moderated mediation model results for Time 1 showed that the interaction between N170 amplitude and age on reading was marginally significant (β = –0.01, *p* = .079, 95 % CI [–0.03, 0.01]), with significant direct effects at ages 10.6 (b = 0.291, *p* = .005, 95 % CI [0.090, 0.492]) and 12.1 (b = 0.310, *p* = .040, 95 % CI [0.090, 0.492]) but not at 8.6 years (*p* = .112). Similarly, at Time 2, the interaction between N170 amplitude and age on reading was again marginally significant (β = 0.10, *p* = .092, 95 % CI [–0.01, 0.09]), with a significant effect only at the older age of 13.1 years (b = 0.10, *p* = .044) and no significant effects at younger (9.6 and 11.6 years) ages (*ps* >.140).

#### 3.3.2 Longitudinal effects of N170 amplitude of real character across two time periods

The fitting results of the longitudinal predictive model of N170 amplitude for real character on orthographic awareness and reading performance are shown in Fig. 7C. The model shows an acceptable fit, *χ*^2^(27) = 35.187, *p* = 0.134, RMSEA = 0.070, SRMR = 0.070, CFI = 0.972, TLI = 0.955. The model explained 31.0 % of the variance in reading ability at T2. After controlling for autoregressive effects of these variables, the N170 amplitude for real characters at T1 could significantly predict the orthographic awareness (β = 0.215, *p* = 0.023) but not the reading performance (β = 0.024, *p* = 0.723) at T2. Orthographic awareness at T1 significantly predicted the reading performance at T2 (β = 0.277, *p* = 0.010). When included age (T1) as a control variable, the results showed a poor fit, *χ*^2^(35) = 110.300, *p* < 0.001, RMSEA = 0.186, SRMR = 0.217, CFI = 0.764, TLI = 0.650, with only orthographic awareness at T1 could significantly predict the reading performance at T2 (β = 0.28, *p* = 0.048).

Taken together, task-state EEG analysis revealed that N170 amplitude for real, pseudo and false Chinese characters in the character recognition task was not only associated with the orthographic awareness in both testing sessions, but also had predictive value—specifically, N170 at Time 1 predicted orthographic awareness at Time 2. However, the predictive power did not extend to the reading performance at Time 2. Besides, we found that N170 amplitude influenced reading performance across both time points through full mediation by orthographic awareness. However, when age was included as a moderating variable, the moderated mediation model results indicated that the direct effect of N170 amplitude on reading ability strengthens with age, becoming pronounced in later childhood, potentially reflecting the increasing neural specialization for reading as children develop.

## 4 Discussion

This study investigated the neurophysiological mechanisms underlying reading development in school-age children over a year, focusing on rs-EEG power and task-evoked ERP components (N170). Firstly, we observed that rs-EEG power in delta (1–3 Hz), theta (4–7 Haz), alpha (8–12 Hz), and beta (13–30 Hz) bands decreased with age. Results from the character recognition task indicate that the N170 components elicited by both real and pseudo characters showed larger amplitudes compared to those elicited by false characters across both test sessions. Secondly, the rs-EEG power in both theta and delta bands, along with the amplitude of the N170 for real, pseudo, and false characters, was correlated with orthographic awareness and reading performance at both sessions and across sections. Moreover, except for rs-EEG power in delta band, all these neurophysiological measures significantly predicted orthographic awareness in both cross-sectional and longitudinal analyses, but not reading performance. Thirdly, mediation analysis revealed that these neurophysiological measures (rs-EEG power in theta/delta bands and N170 amplitude) predicted reading ability through their impact on children's orthographic awareness. Finally, our findings revealed that some of these pathways were relatively stronger in specific age ranges, suggesting that the influence of neurophysiological activity on reading acquisition is developmentally constrained.

### 4.1 Resting state EEG power and reading ability

The rs-EEG power analysis showed that brain rhythms in both low-frequency (delta and theta) and high-frequency (alpha and beta) bands decreased with ages. These findings of low frequency bands were consistent with many previous studies (Clarke et al., 2001, Meng et al., 2021, Whitford et al., 2007). For example, Whitford et al. (2007) found the absolute power of slow wave (0.5–7.5 Hz), and high frequency (alpha, beta) bands in all brain regions decreased linearly with age, especially in the slow wave frequency band. They suggested that the decrease in EEG power may be due to the elimination of synapses. However, studies on how high-frequency power changes with age have yielded inconsistent results. Some studies found that rs-EEG power in alpha, beta, and gamma bands increased with age (Clarke et al., 2001, John et al., 1980, Tagliazucchi et al., 2012), while others found that alpha band power decreased with age, whereas beta band did not significantly change (Harmony et al., 1995). In addition, Meng et al. (2021) showed an opposite pattern in the development of alpha and gamma band power in 7- to 11-year-old children, with the alpha band increasing but the gamma band decreasing with age. According to Whedon et al. (2020), increase of alpha power with age followed a non-linear pattern, with the rate of increase slowing down between ages 3 and 4. We speculated that one potential reason for previous inconsistent results is the variation in the age of children tested across these studies. Future research may conduct longitudinal studies with school-age children over longer periods to gain more insight into how brain rhythms evolve with maturation and reading development.

Importantly, our data showed significant correlations of delta and theta bands power with the orthographic awareness, character and word recognition at both Time 1 and Time 2. Moreover, SEM results showed that delta and theta bands power could directly predict the orthographic awareness both cross-sectional and longitudinal. This highlights the close relationship between the delta and theta bands activity and orthographic processing, which is fundamental to children’s reading development (Chang et al., 2014, Zarić et al., 2021). Previous studies have demonstrated a close relationship between the delta and theta bands activity and language skills (Schiavone et al., 2014, Tan et al., 2024, Tierney et al., 2014). For example, Schiavone et al. (2014) examined early neurophysiological factors associated with reading difficulties in children and found that normalized spectral amplitude of delta band (0.5–2 Hz) corelated with children's reading fluency, automatic speech and orthography, and rapid naming task performance. Lum et al. (2022) explored the relationship between rs-EEG power and children's language skills. They found a significant negative correlation between theta band power and performance on sentence repetition task, which requires not only the use of phonological, syntactic, semantic processing and language working memory. They suggested that this finding may reflect the association between the maturation of theta oscillations during brain development and language processing abilities.

However, our current study found an association between theta band and orthographic awareness, likely due to the crucial role of orthographic awareness in language processing, primarily involved in orthography processing in language processing. This suggested that the delta and theta bands are not limited to the phonological processing found in previous studies (Goswami, 2011, Meng et al., 2021), but were also associated with orthographic processing. Besides, in study (e.g., Goswami, 2011) of reading difficulties, it has been found that theta and delta oscillations in the brain are crucial for processing speech signals, and individuals with dyslexia have difficulties with the temporal sampling of these low-frequency oscillations. Some studies also found increased delta and theta activity in the rs-EEG of children with dyslexia or reading difficulties (Harmony et al., 1995, Papagiannopoulou and Lagopoulos, 2016). These findings, along with the current study, suggested that these two low frequency neural oscillations may represent fundamental electrophysiological activities that impact reading development.

The current study further expands on previous findings by revealing delta/theta power was significantly associated with reading performance but did not directly predict reading performance. Instead, this relationship was mediated by orthographic awareness. In other words, the significant predictive effect of the rs-EEG power on the reading skills requires the engagement of fundamental components (orthographic processing) in language processing. These results suggest that orthographic knowledge, including letter-sound correspondences, spelling rules, and patterns especially positional rules of characters and words (Conrad and Deacon, 2016, Deacon et al., 2019), plays an essential role in linking brain rhythm and literacy development.

### 4.2 Orthographically related N170 and reading ability

Our result showed that the mean amplitudes of the N170 component elicited by real and pseudo characters were significantly larger than those elicited by false characters at both testing sessions. These findings support our hypothesis and indicate the presence of fine N170 tuning at the group level. Although both types of stimuli were unpronounceable, they differed in orthographic legality: pseudo characters followed the positional rules of Chinese orthography, whereas false characters violated these rules. This sensitivity to orthographic structure suggests that the children had already developed abstract orthographic representations and neural specialization for visual word form processing (Tong et al., 2016, Zhao et al., 2014). This pattern is consistent with previous findings showing stronger N170 responses to orthographically legal stimuli compared to illegal or non-orthographic ones (Cao et al., 2011, Galilee et al., 2024, Tong et al., 2016, Zhao et al., 2014). However, we did not observe a significant N170 amplitude difference between real and pseudo characters at either time point, suggesting that the children had not yet developed sensitivity to the lexicality distinction, which may emerge later with increased reading experience (Eberhard-Moscicka et al., 2015, Eberhard-Moscicka et al., 2016, Tong et al., 2016).

More interestingly, further correlation analyses revealed a negative correlation between N170 amplitudes for all three types of stimuli and orthographic awareness at both Time 1 and Time 2. SEM results further revealed that N170 amplitude at Time 1 could predict orthographic awareness both cross-sectionally and longitudinally. The longitudinal effect was observed only for the N170 amplitude evoked by real characters. Previous studies have consistently shown that learning or reading experience can enhance children's orthographic sensitivity and lead to neural or functional specialization, as evidenced by the emergence of coarse- and fine-N170 tuning following reading acquisition or training (Brem et al., 2010; Zhao et al., 2014; Zhao et al., 2018), supporting the Interactive Specialization (IS) view proposed by Frigo and Johnson (2011). According to this viewpoint, brain function depends on activity/experience, and undergo changes as children learn to read. To step further, reading development and experience related to perceptual expertise in word recognition results in the adaptive changes in the visual word-form system (VWFS), allowing proficient readers to perceive and process words quickly and automatically (Brem et al., 2010). Indeed, our current results indicate that neural tuning or specialization can also predict or promote cognitive activity. More specifically, consistent with previous findings that early N170 amplitude significantly predicts later reading performance in children of alphabetic languages (Bach et al., 2013, Brem et al., 2013), we further found that the N170 amplitude were able to significantly predict Chinese children's orthographic awareness. Taken together, these results highlight a potentially bidirectional relationship between neural specialization and reading development, highlighting the significance of early brain specialization in areas critical to reading acquisition.

Correlational analyses also suggest that the N170 amplitudes for real, pseudo, or false characters were correlated with reading performance (character recognition, word reading and reading fluency). This suggests that the word-related N170 is closely associated with children's reading development and may serve as a reliable neural marker of their reading abilities (Coch and Meade, 2016, Tong et al., 2016, Zhao et al., 2014). It is worth noting that the above-mentioned associations with reading skills are restricted to N170 evoked by real characters. One possible reason is that more complex cognitive processes in visual word recognition is involved in processing real characters. In addition to early visual orthographic processing, reading real characters also involves phonological activation, semantic retrieval, and the mapping of orthography to syllables or semantics (Coltheart et al., 2001, Tan and Perfetti, 1998, Zhang et al., 2016). In contrast, processing pseudo or false characters may not involve such later processing as pseud-character lack meanings semantics, and false character cannot be pronounced. Therefore, the N170 amplitude evoked by real word processing is a more suitable neural marker for assessing children's reading ability.

Similar to the results of rs-EEG power, SEM results showed that the N170 amplitude of real, pseudo and false characters did not directly predict reading skills. Rather, the prediction was realized through the mediation of the orthographic awareness. Convergent evidence has demonstrated the association between brain activities and reading performance in preschoolers and school-age children (Brem et al., 2013, Eberhard-Moscicka et al., 2015, Eberhard-Moscicka et al., 2021, Fraga González et al., 2016, Lui et al., 2020, Tong et al., 2016). However, the current study, for the first time, showed that the effect of N170 neural activity on reading ability was achieved through orthographic awareness as a mediating variable. Taken together with the cognitive implications of N170, orthographic processing may be the underlying mechanism of the association between N170 and children’s reading ability.

In recent years, researchers have attempted to explore the interplay between spontaneous brain activity during rest and basic cognitive skills as well as higher cognitive functions in cross-sectional and longitudinal studies (Broomell et al., 2021, Meng et al., 2021, Whedon et al., 2020). All these studies establish connections between brain activity and cognitive performance through mediating factors, such as basic cognitive skills, which aligns with the current findings. For example, Broomell et al. (2021) found that increased frontal alpha power in children indirectly affects performance in reading and math tests by inhibiting control. This perspective is crucial for understanding the relationship between brain activity and behavior, as it emphasizes the hierarchical influence of mediating factors. Identifying these mediators can help uncover the mechanisms by which the brain supports individuals in performing various cognitive tasks.

### 4.3 Potential influence of age

The current study included participants with a relatively wide age range, reflecting the developmental variability in reading-related neural and behavioral processes. The developmental trajectories of both rs-EEG and task-state ERP measures highlight the crucial role of age in shaping neural and cognitive processes. Specifically, both rs-EEG power and N170 amplitude exhibited age-related decreases. Furthermore, the predictive relationships between these neural measures, orthographic awareness, and reading performance appeared to be moderated by age.

The effect of orthographic awareness on the reading performance remains significant even if the factor of age was controlled in the analysis. This aligns with the findings of previous behavioral studies (Cao et al., 2011, Conrad et al., 2013, Zou et al., 2019), demonstrating the importance of the orthographic awareness in Chinese children’s reading development. However, after controlling for age, delta and theta power no longer predicted orthographic awareness, suggesting that age-related maturation may underlie these associations. A similar pattern was reported by Kwok et al. (2019), where the link between alpha power and language performance disappeared once age was controlled. This may be due to the fact that rs-EEG power is closely related to brain maturation in children, resulting a significant correlation with age. Nevertheless, the moderated mediation analysis revealed clear age-dependent effects of both delta and theta power on orthographic awareness and reading ability. Specifically, these neural markers had stronger predictive effects of reading performance or orthographic awareness in younger children, whereas their influence diminished or even reversed with increasing age. This pattern suggests that low-frequency neural oscillations may serve as a key neural mechanism underlying early stages of reading-related cognitive development. Previous research has shown that the decline in slow-wave power is associated with a reduction in gray matter (Whitford et al., 2007). Therefore, one possible explanation for this age-dependent effect is that the normal maturation of the brain provides the foundation for the development of both basic and higher cognitive functions in children.

Similarly, the SEM results showed that the predictive effect of N170 amplitude on the orthographic awareness was no longer significant after controlling for age. Likewise, some previous research with a wide age range of participants (Brem et al., 2009) also found that word-specific N170 decreased with age after controlling for the reading performance, and that the impact of reading skills on N170 was not significant after age was controlled. According to Brem and colleagues (2009), the decreasing N170 amplitude with age, observed in this and other studies, likely reflects the progressive neural tuning and efficiency associated with reading expertise rather than a universal maturation. Supporting this interpretation, our moderated mediation analysis revealed an interaction between age and N170 amplitude in predicting reading performance: among older children, lower N170 amplitudes were associated with improved reading outcomes. This finding suggests that the predictive power of the N170 component may emerge only after a certain level of reading-related neural specialization has been established. In other words, the N170 becomes a meaningful neural marker of reading ability only when sufficient experience and functional refinement have shaped the underlying neural circuits.

A key contribution of our findings lies in the contrasting age-related patterns observed between resting-state and task-evoked neural activity. Resting-state EEG power (delta and theta) was more strongly associated with reading outcomes in younger children, whereas task-evoked N170 amplitude showed a stronger relationship in older children. This dissociation may indicate a developmental shift in the neural mechanisms underlying reading. In early stages, resting-state oscillations reflect general brain maturation and neurocognitive readiness (Beese et al., 2017, Lum et al., 2022, Mantini et al., 2007), which serve as foundational processes for learning to read. As reading experience accumulates, task-evoked neural responses such as the N170 begin to serve as more robust predictors of reading performance, reflecting enhanced neural efficiency and specialization (Huo et al., 2025, Zhao et al., 2019). Together, these findings point to a developmental reallocation of neural resources—from broad, non-task-specific brain maturation that supports early acquisition to more specialized, task-evoked processing associated with skilled reading.

### 4.4 Limitations and future directions

Despite its contributions, the present study has several limitations that should be acknowledged. First, the sample size within each grade was relatively small, which may limit the generalizability of the findings and reduce the sensitivity to detect fine-grained developmental trajectories in brain–behavior associations. Future research should consider recruiting larger samples with narrower age ranges within each developmental stage to minimize age-related variability. Moreover, adopting longer-term or multi-phase longitudinal designs—spanning several years or capturing multiple developmental stages—would allow for a more comprehensive understanding of the dynamic interplay among general brain maturation, neural specialization, and reading development.

Second, although we examined resting-state EEG power in conventional frequency bands, we did not distinguish between periodic (oscillatory) and aperiodic components of the EEG signal. As recent studies have emphasized the functional relevance of both components (Caffarra et al., 2024, Cellier et al., 2021, Chyl et al., 2021), future work incorporating spectral parameterization methods may provide a more nuanced understanding of the neural mechanisms underlying reading acquisition.

Finally, while the current findings highlight the importance of orthographic awareness in mediating the relationship between brain activity and reading ability, further research is needed to explore how targeted educational interventions—particularly those aimed at enhancing sensitivity to orthographic regularities—may accelerate reading development. Understanding how neurocognitive insights can inform instructional practices remains a promising direction for future applied research.

## 5 Conclusion

In conclusion, this longitudinal study examined the relationship between brain activity and reading development in children aged 7–13 years. We found that rs-EEG power and N170 amplitudes decreased with age, reflecting neural maturation. The N170 amplitudes elicited by real and pseudo characters were larger than those for false characters, indicating fine-tuned sensitivity to orthographic structures. Importantly, rs-EEG power and N170 amplitudes directly predicted orthographic awareness, which mediated their effect on reading performance. Moreover, by explicitly modeling the moderating role of age, the present study extends prior research and offers novel evidence that the relationship between neural measures and reading outcomes is developmentally dynamic. These findings underscore the complementary roles of resting-state EEG and task-evoked ERP components, with the former capturing domain-general neural readiness and the latter reflecting domain-specific specialization. This integration offers insights into how general neural maturation supports reading development and has potential for developing biomarkers to identify children at risk of reading difficulties.

## CRediT authorship contribution statement

**Taomei Guo:** Writing – review & editing, Validation, Supervision, Project administration, Investigation, Funding acquisition, Formal analysis, Conceptualization. **Zeping Liu:** Writing – review & editing, Writing – original draft, Validation. **Man Zhang:** Writing – review & editing, Writing – original draft, Visualization, Validation, Methodology, Investigation, Formal analysis, Data curation, Conceptualization. **Pengfei Lu:** Visualization, Conceptualization. **Xuedi Liu:** Writing – review & editing, Visualization. **Li Liu:** Methodology, Conceptualization.

## Funding

This study has been supported by the scientific and technological innovation 2030 - the major project of the Brain Science and Brain-Inspired Intelligence Technology (2021ZD0200500). The funders had no role in study design, data collection and interpretation, or the decision to submit the work for publication.

## Declaration of Competing Interest

The authors declare no conflict of interest for the present study.

## Data Availability

Data will be made available on request.
